# Effect of the COVID-19 Pandemic Lockdown on the Management of Diabetic Retinopathy: A Cross-Sectional Study

**DOI:** 10.7759/cureus.27623

**Published:** 2022-08-02

**Authors:** Vaishali Prajapati, Krishna Shah, Dhruvi Shah, Mayur B Wanjari, Deepika Singhal

**Affiliations:** 1 Ophthalmology, GMERS (Gujarat Medical Education & Research Society) Medical College and Civil Hospital, Ahmedabad, IND; 2 Research, Jawaharlal Nehru Medical College, Datta Meghe Institute of Medical Sciences (Deemed to be University), Wardha, IND

**Keywords:** non-proliferative diabetic retinopathy (npdr), covid-19 fallout, etdrs classification, npdr, csme, diabetic retinopathy, covid-19

## Abstract

Introduction

Worldwide, diabetic retinopathy (DR) is one of the leading causes of vision loss. Early treatment and screening for DR have a major role in reducing the rate of the disease and the coronavirus disease 2019 (COVID-19) pandemic-related restrictions have altered real-world practice patterns in managing DR.

Aims and objectives

To evaluate the impact of the COVID-19 pandemic on the management of DR amongst patients presenting to a tertiary eye care center in Gujarat, India.

Methods

This is a cross-sectional study comparison of ophthalmic findings of 72 patients who presented to a tertiary care hospital with the manifestation of DR before and after the COVID-19 outbreak and subsequent lockdown. All the patients underwent detailed ophthalmic examinations, including optical coherence tomography (OCT) and fundus fluorescein angiography (FFA).

Results

The mean age of participants was 54.5 years, with the mean duration of diabetes being five years since first detected. Diabetes was present in 26 patients out of 72. The number of follow-up visits to an ophthalmologist before COVID-19 was at least every one to three months, which significantly decreased after the lockdown of COVID-19. We found a significant progression of DR and clinically significant macular edema (CSME) in patients with diabetes. Before COVID-19, there were two mild non-proliferative diabetic retinopathy (NPDR), seven moderate NPDR, 15 severe NPDR, and 15 very severe NPDR, which were increased post lockdown to three, nine, 27, and 21, respectively. The proliferative diabetic retinopathy (PDR) vitreous hemorrhage (VH) and tractional retinal detachment (TRD) were also increased to 12 after lockdown as compared to only six before the COVID-19 lockdown. The causes for progression are inability to attend regular check-ups, inability to take proper treatment of diabetes and DR, poor control of diabetes, episode of COVID-19, history of high dose of steroid use, poor kidney function, and not knowing that there is a progression of the disease. A common reason for not visiting an ophthalmologist was fear of the unknown due to COVID-19.

Conclusions

COVID-19 has severely impacted the routine follow-up of DR and, in the subsequent years, there might be an increased incidence of severe outcomes due to DR. The second wave of COVID-19 and its lockdown have had very significant effects on the visual outcome of untreated DR patients.

## Introduction

Since the outbreak of coronavirus disease 2019 (COVID-19) in March 2020 and the subsequent lockdown, the health system was geared toward COVID-19, and there was relative neglect towards care for non-communicable diseases. Progression of a few non-communicable diseases is very rapid and, if interventions are not done, can lead to severe complications. One such is diabetic retinopathy (DR), which requires timely intervention to prevent severe outcomes. There are various factors and poor glycaemic control for visual morbidity due to DR [1,2].

DR is one of the leading causes of blindness. It is a microvascular complication of diabetes mellitus due to which diabetic macular edema, as well as proliferative diabetic retinopathy, occurs. Both conditions need necessary and emergent treatment and a regular follow-up [3,4].

As shreds of evidence from critical clinical trials, it is proved that anti-vascular endothelial growth factor injections are the standard treatment for caring for diabetic macular edema [5-6]. Common treatments include pan-retinal photocoagulation (PRP) and anti-vascular endothelial growth factor (VEGF) injections, as well as a combination of the two for treating proliferative diabetic retinopathy (PDR) [3-7]. Early intervention might be useful before the DR progression to later stages of complications, such as vitreous hemorrhage (VH) and tractional retinal detachment (TRD) [8-10]. Amongst patients diagnosed with DR, complications might lead to worsening the outcome and leads to irreversible vision loss. 

## Materials and methods

A cross-sectional study comparison of ophthalmic findings of 72 patients who presented to a tertiary care hospital, GMERS Medical College and Civil Hospital, Ahmedabad, Gujarat, India, with the manifestation of DR before and after the COVID-19 outbreak and subsequent lockdown. All the patients were undergoing detailed ophthalmic examinations, including optical coherence tomography (OCT) and fundus fluorescein angiography (FFA) as and when required. All the patients were defined by the standard Early Treatment Diabetic Retinopathy Study (ETDRS) classification of DR. All patients were compared with their previous ophthalmic examination records. The disease progression was compared and documented along with the biochemical markers of diabetes and history of COVID-19 infection. The delay and reasons for the delay in reaching out to the ophthalmologist for follow-up were also documented.

Statistical analysis

We used IBM SPSS Statistics for Windows, Version 23.0 (Released 2015; IBM Corp., Armonk, New York, United States) for the analysis of the result of this study. Demographic data were collected by the questionnaire, which was developed by the researcher. A descriptive statistical method was used to analyze the data.

Ethical approval 

The ethical approval was taken from the Institutional Committee, GMERS Medical College and Civil Hospital, Ahmedabad, Gujarat, India (GMERSMCS/IEC/40/2021).

## Results

The mean age was 54.5 years with a minimum of 33 years and the maximum was 76 years of age. The age distribution maximum was 41-60 years in both males and females, 23 (31.95%) and 30 (41.66%), respectively (Table 1).

**Table 1:** Age distribution

Age Group (Years)	Male	Female	Total
30-40	3 (4.16%)	4 (5.56%)	7 (9.72%)
41-50	12 (16.67%)	13 (18.05%)	25 (34.72%)
51-60	11 (15.28%)	17 (23.61)	28 (38.89%)
61-70	5 (6.94%)	4 (5.56%)	9 (12.50%)
>70	1 (1.39%)	2 (2.78)	3 (4.17%)
Total	32	40	72

The mean duration of diabetes was five years, with a maximum of recently diagnosed in 12 (16.67%) males and 14 (19.44%) females. The recently diagnosed were a maximum of 26 (36.11%) while there were six (8.34%) with more than 10 years of diabetes (Table 2).

**Table 2:** Duration of diabetes

Duration	Male	Female	Total
Recently Diagnosed	12 (16.67%)	14 (19.44%)	26 (36.11%)
1-3 years	4 (5.56%)	5 (6.94%)	9 (12.5%)
3-5 years	5 (6.94%)	7 (9.72%)	12 (16.66%)
5-7 years	3 (4.17%)	4 (5.56%)	7 (9.73%)
7-10 years	5 (6.94%)	7 (9.72%)	12 (16.66%)
>10 Years	3 (4.17%)	3 (4.17%)	6 (8.34%)
Total	32	40	72 (100%)

There has been a significant increase in the severity of diabetic retinopathy after lockdown. Out of 72 patients, 60 (83.34%) patients developed NPDR changes and 12 (16.66%) patients developed PDR changes, which are very significant (Table 3).

**Table 3:** Diabetic retinopathy manifestation before and after COVID-19 lockdown NPDR: non-proliferative diabetic retinopathy;  PDR: proliferative diabetic retinopathy; VH: vitreous hemorrhage; TRD: tractional retinal detachment; COVID-19: coronavirus disease 2019

Diabetic Retinopathy Manifestation	Male		Female		Total	
	Before	After	Before	After	Before	After
Mild NPDR	1 (1.39%)	2 (2.78%)	1 (1.39%)	1 (1.39%)	2 (2.78%)	3 (4.17%)
Moderate NPDR	4 (5.55%)	5 (6.94%)	3 (4.17%)	4 (5.55%)	7 (9.72%)	9 (12.5%)
Severe NPDR	6 (8.33%)	11 (15.28%)	9 (12.50%)	16 (22.22%)	15 (20.83%)	27 (37.50%)
Very severe NPDR	7 (9.72%)	9 (12.50%)	8 (11.11%)	12 (16.67%)	15 (20.83%)	21 (29.17%)
PDR (VH and TRD)	2 (2.78%)	5 (6.94%)	4 (5.55%)	7 (9.72%)	6 (8.33%)	12 (16.66%)
Total	20/32	32/32	25/40	40/40	45/72	72/72

Diabetes-related retinal complications such as CSME increased dramatically after the COVID-19 lockdown. Of the total of 72 patients, 17 (23.61%) were diagnosed with CSME after the COVID-19 pandemic (Table 4).

**Table 4:** Clinically significant macular edema before and after the COVID-19 lockdown CSME: clinically significant macular edema; COVID-19: coronavirus disease 2019

Presentation of CSME	Male		Female	
	Before	After	Before	After
CSME	10 (13.88%)	18 (25%)	18 (25%)	27 (37.50%)
No CSME	22 (30.56%)	14 (19.44%)	22 (30.56%)	13 (18.05%)
Total	32	32	40	40

Drop-in best-corrected visual acuity has been seen after COVID-19 lockdown due to the progression of DM manifestations. The mean was 6/24 while the maximum drop-in visual acuity was noted as6/24-6/60 in 27 (37.50%) out of 72 patients (Table 5). 

**Table 5:** Visual acuity on Snellen's chart

Visual Acuity on Snellen's Chart	Male		Female		Total	
	Before	After	Before	After	Before	After
6/6-6/12	5 (6.94%)	3 (4.17%)	7 (9.72%)	5 (6.94%)	12 (16.66%)	8 (11.11%)
6/12P-6/24	10 (13.89%)	7 (9.72%)	10 (13.89%)	9 (12.50%)	20 (27.78%)	16 (22.22%)
6/24P-6/60	13 (18.06%)	12 (16.67%)	18 (25%)	15 (20.83%)	31 (43.05%)	27 (37.51%)
<6/60	4 (5.56%)	10 (13.89%)	5 (6.94%)	11 (15.28%)	9 (12.50%)	21 (29.16%)
Total	32	32	40	40	72	72

Though many factors are responsible for the progression of DR manifestations, two significant factors were poor control of diabetes mellitus (87.50%) and the inability to contact an ophthalmologist due to the COVID-19 pandemic (86.11%) (Table 6).

**Table 6:** Associated factors in DR manifestation DR: diabetic retinopathy; COVID-19: coronavirus disease 2019

FACTORS	Male	Female	Total
Previous history of DR Treatment	26 (36.11%)	33 (45.83%)	59 (81.94%)
Poor Control of diabetic mellitus	27 (37.5%)	36 (50%)	63 (87.50%)
Uncontrolled blood pressure	25 (34.72%)	29 (40.28%)	54 (75%)
Altered kidney function	22 (30.55%)	25 (34.72%)	47 (65.27%)
Increased lipid profile	7 (9.72%)	8 (11.11%)	15 (20.83%)
COVID-19 episode	19 (26.39%)	27 (37.50%)	46 (63.89%)
History of steroid use	15 (20.83%)	22 (30.55%)	37 (51.38%)
Poor economic condition	24 (33.33%)	34 (47.22%)	58 (80.55%)
Not able to contact an ophthalmologist	29 (40.28%)	33 (45.83%)	62 (86.11%)

## Discussion

COVID-19 has spread across more than 210 countries and territories around the world. Since the outbreak of COVID-19 and subsequent lockdown, the health system was geared toward COVID-19, and there was relative neglect towards care for non-communicable diseases. Progression of a few non-communicable diseases is very rapid and, if interventions are not done, can lead to severe outcomes. One such is DR, which requires timely intervention to prevent severe outcomes. There are various factors and poor glycemic control for visual morbidity due to DR.

The majority of diabetic patients are above 45 years of age. In the COVID-19 pandemic, people with comorbid conditions were more severely affected than others. The lockdown and the fear of getting infected by the COVID-19 virus have led to many losses to follow-up patients. Uncontrolled diabetes in the long term leads to microangiopathic changes in the body; diabetic retinopathy is one of them [11]. If not treated early, the prognosis is poor.

Although scary, most of the published evidence suggests that delay or ignorance of other non-communicable diseases in the COVID-19 pandemic has impacted the rapid worsening of these diseases [8]. DR, amongst them, has become one of the leading causes of progressive, irreversible blindness in specific age groups.

In the present study of 72 diabetic patients, the mean visual acuity decreased from 6/24 before the lockdown to 6/36 after the lockdown. In a similar study, Saleh [1] found that out of 132 patients, the mean visual acuity significantly decreased from 6/18 before the lockdown to 6/24 after the lockdown.

In the present study, out of 72 patients, 28 had developed CSME-induced low visual acuity before COVID-19, while 45 patients developed significant visual acuity loss after the COVID-19 lockdown.

Chatziralli et al. [12] conducted a similar study in which they noted that out of 62 patients with CSME, 11 had very low visual acuity before COVID-19 lockdown while 21 patients presented very low acuity after the COVID-19 lockdown.

There is a significant complication in the eyes as a long-term sequel due to unmanaged diabetes. The research revealed that the diabetic mellitus to diabetic retinopathy disease process takes 15 years. The leading cause of blindness is the DR in developed countries [13]. Across a spectrum, PDR changes to NPDR occur due to the changes in the eye span. These changes can be subsided by regular follow-up and ensuring that glucose level is in control, checking of blood pressure, and checking of eyes. Several treatments, including laser photocoagulation, injecting steroids, anti-vascular endothelial growth factor, or vitrectomy, can be used to manage the various stages of these eye changes [14].

Due to the lockdown, the patient could not attend a clinic previously diagnosed with DR and diabetic macular edema for which regular follow-ups were essential, and intravitreal injection appointments were delayed. The result was found to be significant that was seen worsening in best corrected visual acuity (BCVA) and central retinal thickness (CRT) in patients with diabetic macular edema, as well as in progression to active PDR in 30% of patients with severe NPDR and 8.3% of patients with previously quiescent PDR. As diabetic patients were found to exhibit a small which seemed to be independent of glycemic control and significant improvement in glycemia, body weight, and total cholesterol, and the other parameters were found to be stable [15].

Ongoing and current treatment is anti-VEGF for patients diagnosed with DME. The study findings show significant visual loss occurs when suspending intravitreal injection appointments or follow-up visits in patients with DME [16]. The treatment should be received at the earliest to avoid the devastating complications of PDR. Since our results showed that delays in treatment were associated with a change in BCVA, prolonged treatment postponement and indefinite deferral of the appointments without rescheduling within a reasonable time should be avoided, depending on measures in each country [16].

Limitations of the study

The limitations of the study are its short duration, the retrospective study design, and the low number of participants.

## Conclusions

COVID-19 has severely impacted the routine follow-up of DR, and in the subsequent year, there might be an increased incidence of severe outcomes due to DR. The second wave of COVID-19 and its lockdown had very significant effects on the visual outcome of untreated DR patients
